# Evaluation on the effect of acupuncture on patients with sepsis-induced myopathy (ACU-SIM pilot study): A single center, propensity-score stratified, assessor-blinded, prospective pragmatic controlled trial

**DOI:** 10.1097/MD.0000000000020233

**Published:** 2020-05-22

**Authors:** Wei-Tao Chen, Ce Sun, Ying-Bin Zhou, Dong-Hua Liu, Zhi-Long Peng, Jing Chen, Nuo Xu, Yan-Yan Lei, Jun He, Chun-Zhi Tang, Xin-Feng Lin, Neng-Gui Xu, Shao-Xiang Xian, Li-Ming Lu

**Affiliations:** a Intensive Care Unit, The First Affiliated Hospital of Guangzhou University of Chinese Medicine; b Ling-Nan Medical Research Center, Guangzhou University of Chinese Medicine; c The First Clinical School of Guangzhou University of Chinese Medicine, Guangzhou; d Meizhou Hospital of Traditional Chinese Medicine, Meizhou; e Department of Acupuncture and Rehabilitation, The First Affiliated Hospital of Guangzhou University of Chinese Medicine, Guangzhou, China; f The university of Alabama at Birmingham, Birmingham, USA; g Medical College of Acupuncture and Rehabilitation; h Guangzhou University of Chinese Medicine; i The First Affiliated Hospital of Guangzhou University of Chinese Medicine; j School of economics and management, Guangzhou University of Chinese Medicine, Guangzhou, China.

**Keywords:** acupuncture, muscle wasting, pragmatic controlled trial, sepsis

## Abstract

**Background:**

Sepsis-induced myopathy (SIM) is a disease that causes motor dysfunction in patients with sepsis. There is currently no targeted treatment for this disease. Acupuncture has shown considerable efficacy in the treatment of sepsis and muscle weakness. Therefore, our research aims to explore the effects of acupuncture on the improvement of muscle structure and function in SIM patients and on activities of daily living.

**Methods:**

The ACU-SIM pilot study is a single-center, propensity-score stratified, assessor-blinded, prospective pragmatic controlled trial (pCT) with a 1-year follow-up period. This study will be deployed in a multi-professional critical care department at a tertiary teaching hospital in Guangzhou, China. Ninety-eight intensive care unit subjects will be recruited and assigned to either the control group or the acupuncture group. Both groups will receive basic treatment for sepsis, and the acupuncture group will additionally receive acupuncture treatment. The primary outcomes will be the rectus femoris cross-sectional area, the Medical Research Council sum-score and time-to-event (defined as all-cause mortality or unplanned readmission to the intensive care unit due to invasive ventilation). The activities of daily living will be accessed by the motor item of the Functional Independence Measure. Recruitment will last for 2 years, and each patient will have a 1-year follow-up after the intervention.

**Discussion:**

There is currently no research on the therapeutic effects of acupuncture on SIM. The results of this study may contribute to new knowledge regarding early muscle atrophy and the treatment effect of acupuncture in SIM patients, and the results may also direct new approaches and interventions in these patients. This trial will serve as a pilot study for an upcoming multicenter real-world study.

**Trial registration:**

Chinese Clinical Trials Registry: ChiCTR-1900026308, registered on September 29th, 2019.

## 1 Introduction

Rapid muscle wasting is commonly seen in septic patients during their very first days within the intensive care unit (ICU).^[1,2]^ This phenomenon has been described as sepsis-induced myopathy (SIM),^[3,4]^ one of the most vital components of ICU-acquired weakness.^[4]^ This persistent situation is more common in skeletal muscles and respiratory muscle groups.^[5,6]^ If left untreated, or even the intervention is just delayed, SIM could last for years in survivors of sepsis. SIM can lead to prolonged mechanical ventilation, long-lasting limb motor dysfunction, a protracted in-hospital stay, unplanned readmission and increased long-term mortality.^[7,8]^

At present, there is no targeted treatment for SIM. The efficacy of early rehabilitation (such as in-bed passive/active leg cycling therapy) remains controversial.^[2,9]^ Initiation of these kinds of intervention has barriers in the real world (ie, lack of facilities, personnel and equipment, or the patient cannot fully cooperate with the muscle group exercises).^[10]^ In addition, septic patients may need central venous catheterization and/or invasive arterial monitoring (A-line), not to mention the needs of those who received Intra-aortic balloon pump or extracorporeal membrane oxygenation. All of these factors lead to difficulties in conducting early physical therapy in septic patients. Hence, the development of an easy-to-perform, SIM-targeted treatment is of paramount importance for improving muscle wasting in patients with sepsis.

Acupuncture has been shown to have some advantages in patients with sepsis or muscular diseases separately. However, to the best of our knowledge, no study of acupuncture has been performed or is ongoing in patients with sepsis combined with muscle wasting.

First, several animal experiments have confirmed that acupuncture can improve sepsis by regulating inflammation. Scognamillo-SzabÓ and his colleagues reported that acupuncture can reduce the degree of inflammation during sepsis and effectively curb the development of sepsis.^[11]^ In an LPS-stimulated rat model, pretreatment with acupuncture at ST36 (Zusanli) in septic mice reduced the degree of kidney damage by lowering iNOS levels.^[12]^ In addition, Song JG et al indicated that electroacupuncture preconditioning increased the survival rate by decreasing the pro-inflammatory cytokine concentration.^[13]^

Second, acupuncture has had therapeutic effects in the treatment of acute muscle wasting. Su and his teammates found that acupuncture was effective against skeletal muscle atrophy and promoted muscle regeneration in denervation-induced muscular atrophic mice.^[14]^ Suzuki et al revealed that acupuncture significantly improved grip strength and respiratory muscle strength in chronic obstructive pulmonary disease participates within a RCT.^[15]^ After 12 weeks of treatment, the improvements in the grip strength and respiratory muscle strength in the acupuncture group were significant when compared to those in the sham-acupuncture group. This indicates that acupuncture might have an effect on the improvement of muscle weakness.^[15]^ In addition, Takaoka et al found that acupuncture can alleviate muscle atrophy in rats by downregulating the gene expression of myostatin (MSTN) in an animal experiment.^[16]^

Electrical muscle stimulation (EMS) seems to be another candidate for treating muscle weakness. Several studies have already suggested that neuromuscular electrical stimulation might have potential benefits in a rat model of acute muscle atrophy.^[17,18]^ Furthermore, researchers conducted a clinical RCT that enrolled 140 ICU patients into two arms, 68 into the EMS arm and 72 into the control arm; daily EMS treatment was provided in the EMS arm. The results showed that the incidence of polyneuromyopathy, a critical illness, was lower in the EMS arm than in the control arm, indicating that conventional EMS can attenuating the development of muscle weakness in ICU patients.^[19]^ It is a pity that this trial was not specifically designed for septic patients. In summary, based on the results of the existing literature, a therapeutic effect of EMS on sepsis has not yet been shown.

There is currently no clinical evidence, either randomized controlled trials or real-world studies, for acupuncture treatment of SIM. Our group intends to analyze prospective data through real-world research methods and to explore the clinical effects of acupuncture on septic muscle atrophy to provide a basis for subsequent clinical research.

Based on the clinical instinct and the experience of our team, septic patients experienced acupuncture treatments during their ICU stays. We hope that this study will provide some references for the upcoming clinical trial.

### 1.1 Research objectives

Acupuncture has already been deployed in the treatment of sepsis or muscle weakness. Nevertheless, its efficacy and safety in SIM remains unclear. Therefore, this study has the following research objectives:

(1) To describe the progression of loss of muscle mass (the change of rectus femoris cross-sectional area [RF_CSA]_ from baseline) in patients with sepsis after admission to the ICU and to investigate whether the decline in RF_CSA_ is the morphological basis for muscle weakness in septic patients.
(2) To assess whether acupuncture at the Yang-Ming meridian acupoints affects the changes in RF_CSA_ and muscle strength and the long-term prognosis and safety in SIM simultaneously.
(3) To serve as pilot research for an upcoming multicenter real-world study.

## 2 Methods

### 2.1 Design

The ACU-SIM pilot study is a single center, propensity-score stratified, assessor-blinded, prospective pragmatic controlled trial with a 1-year follow-up period. We hope to assess the therapeutic effect and safety of acupuncture on SIM patients.

### 2.2 Setting

This study will be implemented in a 37-bed interdisciplinary critical care ward of a tertiary, academic, teaching hospital: the first affiliated hospital of Guangzhou University of Chinese Medicine, China. All diagnoses and treatments will be performed by trained physicians and technicians.

### 2.3 Sample size

Temporarily, there are no data on how acupuncture benefits patients with SIM. Therefore, we searched for related studies. In the trial “Electrical muscle stimulation prevents critical illness polyneuromyopathy.”^[19]^ the incidence rates were 12.5% in the EMS group and 39.3% of the control group. Assuming α = 0.05 and 1-β = 0.8, and an experimental group to control group ratio of 1:1, the sample size was calculating by STATA software (STATA IC 15.0, Stata Corp, College Station, TX). We will need 39 patients in the experimental group and 39 patients in the control group. Considering factors such as dropouts, the sample size of the acupuncture group and the control group is designed to be 49 and 49, respectively; a total of 98 cases will be observed.

### 2.4 Study procedures

#### 2.4.1 Electronic data capture (EDC) System

The Department of Critical Care Medicine at the First Affiliated Hospital of Guangzhou University of Traditional Chinese Medicine has already deployed a cloud-based EDC system (website: https://study.empoweredc.com/site/203). Each patient will be assessed within 24 hours after ICU admission, and patients who meet the sepsis diagnosis will be entered into the system. Peripheral serum will be collected prospectively at baseline and at subsequent time points and will be stored in the biological sample bank (Ling-Nan Medical Research Center) to test MSTN expression levels. Patients who meet the SIM diagnosis will be marked in the EDC according to the diagnostic criteria. Two intensivists will measure the RF_CSA_ of the sepsis patients at the measurement time point and will then enter the data into the EDC system; a consistency evaluation will be performed. One ICU specialist and 1 neurologist will evaluate the medical research council sum-score (MRC-SS) of the SIM patients.

#### 2.4.2 Participant eligibility

**Figure 1:**
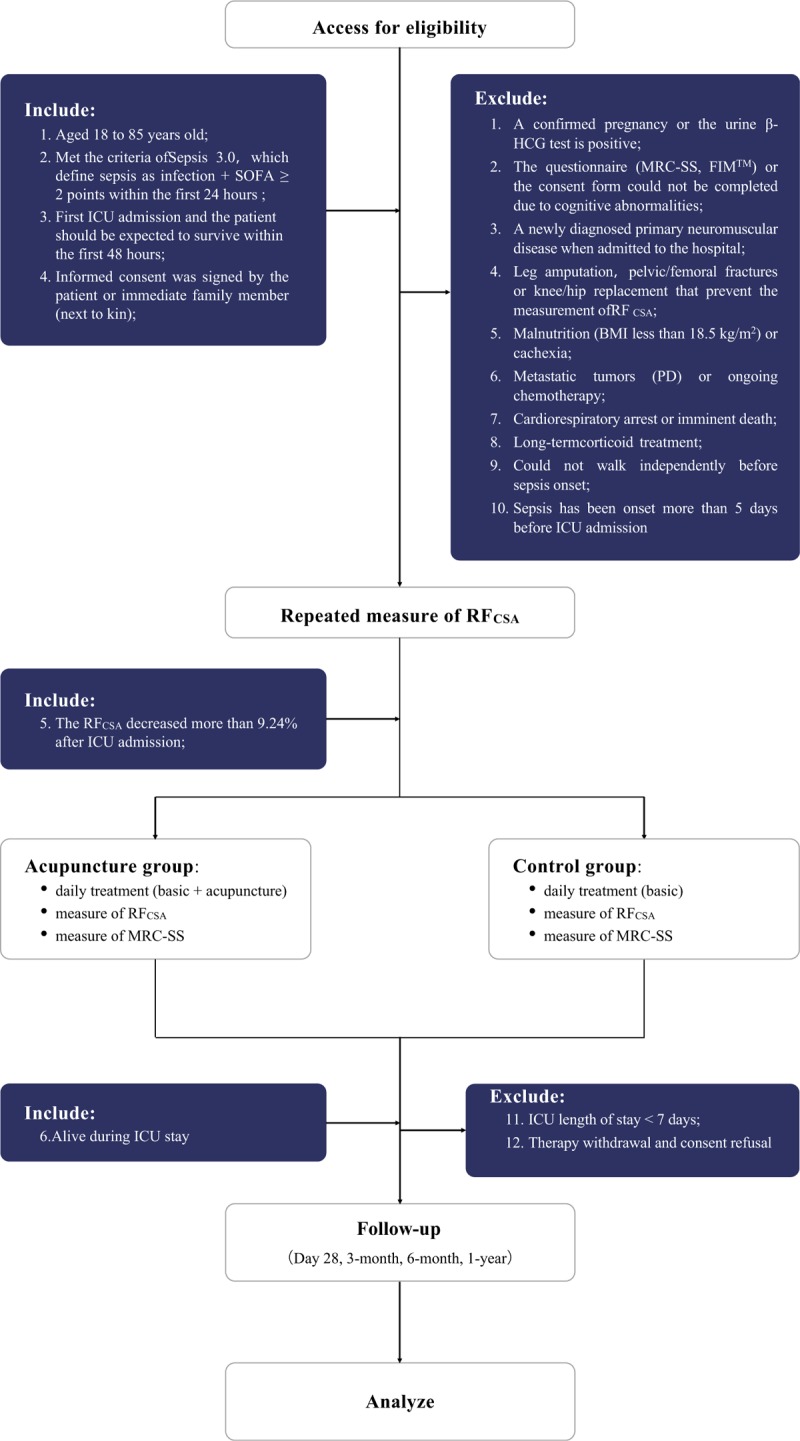
Study flowchart.

A total of 98 subjects will be recruited from our multidisciplinary ICU if they fulfill the inclusion criteria and do not have any exclusion criteria. Figure 1 shows the overview of the recruitment process.

#### 2.4.3 Inclusion criteria

(1) Age 18 to 85 years old;
(2) Met the criteria of Sepsis 3.0^[20]^, which defines sepsis as infection + sequential (sepsis) organ failure assessment score (SOFA) ≥ 2 points within the first 24 hours;
(3) First ICU admission and the patient should be expected to survive within the first 48 hours;
(4) Informed consent was signed by the patient or their next to kin;
(5) The RF_CSA_ decreased more than 9.24%^[21]^ after ICU admission;
(6) Alive during the ICU stay.

#### 2.4.4 Exclusion criteria^[1,2]^

(1) A confirmed pregnancy or the urine β-HCG test is positive;
(2) The questionnaires (MRC-SS and FIM^TM^) or consent form could not be completed due to cognitive abnormalities.
(3) A newly diagnosed primary neuromuscular disease when admitted to the hospital;
(4) Leg amputation, pelvic/femoral fractures or knee/hip replacement that prevent the measurement of the RF_CSA_;
(5) Malnutrition (BMI less than 18.5 kg/m^2^) or cachexia;
(6) Metastatic tumors (PD) or ongoing chemotherapy;
(7) Cardiorespiratory arrest or imminent death;
(8) Long-term corticoid treatment;
(9) Could not walk independently before sepsis onset;
(10) ICU length of stay (LOS) < 7 days^[2]^;
(11) Sepsis onset more than 5 days before ICU admission^[2]^;
(12) Therapy withdrawal or consent refusal.

Patients are eligible if they are diagnosed with sepsis, their RF_CSA_ decreased more than 9.24%^[21]^ from baseline, and informed consent has already acquired from the patient and/or immediate family member. A case report form (CRF) will be completed, and the RF_CSA_, MRC-SS score and other relevant data will be obtained, for all eligible and consenting patients. We will use the RF_CSA_ to assess the changes in muscle morphometry; the MRC-SS will be used to assess whether patients meet the diagnostic criteria of ICU-acquired weakness and to observe mortality, rehospitalization, reintubation and mechanical ventilation and other relevant indicators during the 1-year follow-up.

### 2.5 Intervention

#### 2.5.1 Acupuncture treatment

According to the patient's (and/or their relatives’) willingness and the clinical routine, a group of physicians will assess the acupuncture treatment within 24 hours in patients who meets the inclusion criteria 1 to 5. All physicians are required to have at least 3 years (or 300 hours) of practical experience with acupuncture. The acupuncture treatment will be performed by stainless steel needles (0.30 mm × 40 mm; 0.30 mm × 25 mm; Suzhou Medical Supplies Factory Co., Ltd., Suzhou City, China). The acupuncture treatment will be performed by the technique of lifting, thrusting, twirling and rotating the needle until the patient is being “De-qi” (getting a numbness or other acupuncture feeling). Two points that do not cross the joint will be chosen for electrical stimulation. The needle will be kept in place for approximately 30 minutes. By the time we remove the needles, a clean cotton balls will be pressed to the skin to prevent bleeding.

All patients must be treated at “3 obligatory basic acupuncture points”: ST36, ST40 and LI10. Furthermore, at least 3 additional acupuncture points will be selected according to the principles of traditional Chinese medicine (for example, if the patient has a mental disorder manifestation, acupoints such as GV20, GV24, and EX-HN3 can be added to promote the patient's waking). Additional acupoints could be applied, and ear acupuncture points might also be included. All acupoints selected in each session will be recorded. A traditional Chinese syndrome diagnosis is required, and the diagnosis will be documented. Acupuncture treatment will be performed twice a day and lasts for 30 minutes. If the included subjects do not meet the exclusion criteria and do not terminate their participation in study, they will receive acupuncture treatment during their entire ICU stay. If the patient is discharged from the ICU, the acupuncturist will continue to follow the patient until the 14-day acupuncture session is completed. Both the acupuncture group and the control group will receive basic sepsis treatment. The selection of acupuncture points is listed in Table 1.

**Table 1:**
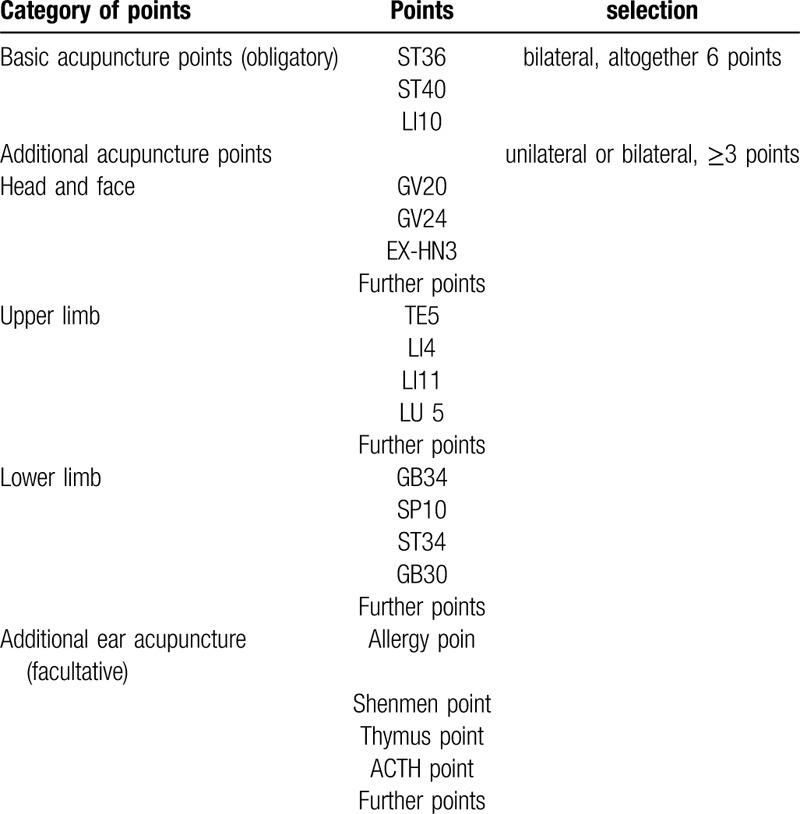
. Selection of acupoints and positioning.

#### 2.5.2 Basic treatment (Standard of care)

The basic treatment was developed based on the "Surviving Sepsis Campaign: International Guidelines for Management of Sepsis and Septic Shock: 2016”.^[22]^ According to those guidelines, targeted blood glucose range will be set between 8-10 mmol/L (90-144 mg/dL),^[23]^ sedative drugs will be ceased as soon as possible, the use of neuromuscular blockers will be restrain, and early physical exercise will be encouraged.^[23]^

### 2.6 Blinding/masking

The acupuncturist will only be responsible for acupuncture treatment. The duration, acupoints, and date of the acupuncture treatments will be recorded in the EDC system when the treatment is finished. The RF_CSA_, MRC-SS and clinical outcome data will not be visible to the acupuncturist.

The RF_CSA_ and MRC-SS measurements will be assessed separately by individual physicians. Another intensivist will perform a spot-check on the RF_CSA_ measurements, and a neurologist will check the MRC-SS measurements at a fixed time point. At the end of the study, a consistency test will be performed on the measurements. Neither the intensivist nor the neurologist will be able to access the measured value of the other assessment, and the assessors will not know the specific acupuncture treatment or other outcome indicators.

Subjects will know their group assignment. Acupuncturists and critical care providers will also know the group assignments of the subjects during treatment. The data analyst will only know that there are 2 groups, but the specific group assignments will be blinded. Because this study is a prospective pragmatic controlled trial (pCT), only the outcome assessors and data analysts will be blinded; blinding will have no effect on the subjects, so there is no need for the study to be unblinded.

### 2.7 Study groups

Since this study is a real-world study, the grouping of the study subjects was determined by the actual observations. Therefore, no randomization of groups was set in this study. The subjects will be as allocated into 2 arms.

Group 1: acupuncture group

When the participant meets the inclusion criteria (1 to 5), the acupuncturist will assess the conditions for patient treatment. Patients can enter this group and have acupuncture treatment if they do not have specific conditions (such as edema, coagulopathy, acupoints local skin and soft tissue infection, and rejection of acupuncture treatment) that are not suitable for acupuncture.

Group 2: control group

Patients who are not suitable for acupuncture or are not willing to receive acupuncture could enter this group. In addition to basic treatment, patients in this group will be prescribed exercises, such as early in-bed activities or wheelchair activities.

### 2.8 Outcome

The primary outcomes will be the decline of RF_CSA,_ the MRC-SS and time-to-event (defined as all-cause mortality or unplanned readmission to the ICU due to invasive ventilation). The activities of daily living will be accessed by the motor item of the Functional Independence Measure (FIM^TM^). Secondary outcome includes the expression level of MSTN, ICU length of stay, length of hospital stay, duration of invasive/non-invasive mechanical ventilation. The outcomes will be measure as follow:

#### 2.8.1 Measurement of RF_CSA_

We will measure the RF_CSA_ on days 1, 3, 5, 7, 10, and 14 by B mode ultrasonography, using a 12L-SC (8.0-13.0 MHz, 7cm) linear array (Venue 50, GE Healthcare) at the First Affiliated Hospital of Guangzhou University of Chinese Medicine. RF_CSA_ will be measured on days 18, 21, 24, 28 if the patient continues to stay in the ICU. Patients will be prone to a 30° upwards incline at the head, except where the clinical conditions dictate otherwise. An excess of gel will be applied to minimize distortion. The transducer should be placed perpendicularly along the superior aspect of the upper leg, two-thirds of the distance between the anterior superior iliac crest and the superior patellar border. RF_CSA_ will be taken as an average of 3 consecutive measurements within 10% of 1 another. (A larger RF_CSA_ indicates a larger muscle mass). The point of measurement will be marked on the skin, and the same limb and location will be measured at each time point.^[1,24]^

#### 2.8.2 Assessment of MRC-SS

Peripheral muscle strength will be measured according to the Medical Research Council sum-score (MRC-SS), which will result in a range of 0 to 60. Higher scores indicate greater muscle strength. Three muscle groups of each extremity will be tested bilaterally: shoulder abductors, elbow flexors, wrist flexors, hip flexors, knee extensors, and ankle dorsiflexors. Muscle weakness will be defined as an MRC-SS less than 48.^[25–27]^ When the patient is in a condition in which the MRC-SS cannot be fully evaluated (eg, 1 or more limb amputations or fractures; the limb cannot move due to indwelling tubing), a mean MRC-SS of less than 4 is used to indicate muscle weakness.^[26]^

Muscle strength will be evaluated at the first awakening and at ICU discharge. The first awakening is defined as the first day when the participant is alert, has a Richmond sedation and agitation scale between -1 and 1 (the Richmond sedation and agitation scale score range from -5 to +4, and -1 to +1 is the ideal score range for full cooperation to the MRC-SS test), and is able to follow at least 3 of the De Jonghe 5-command criteria.^[27]^

#### 2.8.3 Language

This study will include subjects who speak non-Mandarin (including dialects such as Cantonese, Hakka, and Chaoshan and so on). The research team will recruit clinicians who can use the above dialects proficiently to participate in the study. After professional training, these clinicians will be able to explain the research plan and the questionnaire to the subjects in detail. If it is still too difficult for the patient to understand and cooperate because of a language gap, the researcher will explain the relevant content to the patient with the help of the patient's next-of-kin.

#### 2.8.4 Quality of life: FIM^TM^

We will measure physical function by the motor item of the FIM^TM^ at day 28, day 90, six months, and 1 year after the admission to the ICU. The FIM^TM^ is a widely applied scale of functional abilities in rehabilitation patients. This scale can be used to assess a patient's ability to independently perform 13 daily activities (e.g., dressing, toileting, ambulating).^[28,29]^ Higher FIM^TM^ total scores indicate that patients need more help with these activities (score range from 13–91). The change in the FIM^TM^ will then be documented for each participant as the assessment of functional improvement of sepsis survivors.

#### 2.8.5 Collecting clinical samples (blood): MSTN

Concentrations of MSTN will be analyzed in serum using a sandwich-type enzyme-linked immunosorbent assay kit. Samples will be taken on day 1, day 7 and day 14 (or the time of ICU discharge) of the ICU admission. The samples will be stored at -80 °C in the Biomedical Research Library at Ling-Nan Medical Research Center for the assay.

#### 2.8.6 Collecting healthcare data within the ICU stay

During the screening period, the relevant subject information will be completed, and baseline demographic-related items will be collected. The SOFA score, the immediate acute physiology and chronic health evaluation II score (and SAPS score), the GCS score, body temperature, mechanical ventilation or not, the infection site, the pathogenic examination before the administration of antibiotics or use of vasoactive drugs, arterial blood gas analysis, blood routine, coagulation function, liver function, kidney function, heart function, infection index, urine pregnancy test (female subjects), and an electrocardiogram will be recorded during this period. The length of the first antibiotic use and whether the pathogen is sensitive to the antibiotic will be recorded after initiation.

For patients who survive and are discharged from the ICU - meeting all inclusion criteria (1 to 6) - the mechanical ventilation duration time, LOS, total hospital stay, sum sedative analgesic dose, statin dose, blood glucose level, insulin dosage, glucocorticoid dosage, protective constraints, and the duration of neuromuscular blockers will be additionally recorded.

Table 2 shows a general view of the data collecting and the time of acquisition for each item. We will manage all study data anonymously, and all CRFs will be labelled with a study number allocated to each individual participant. The CRF will be in both paper and electronic formats. The paper version of the CRF will be used for backup storage of data and for verification when needed. The daily management of data will be completed on the EDC system. And the details of bedside data collection are shown in Table 3.

**Table 2:**
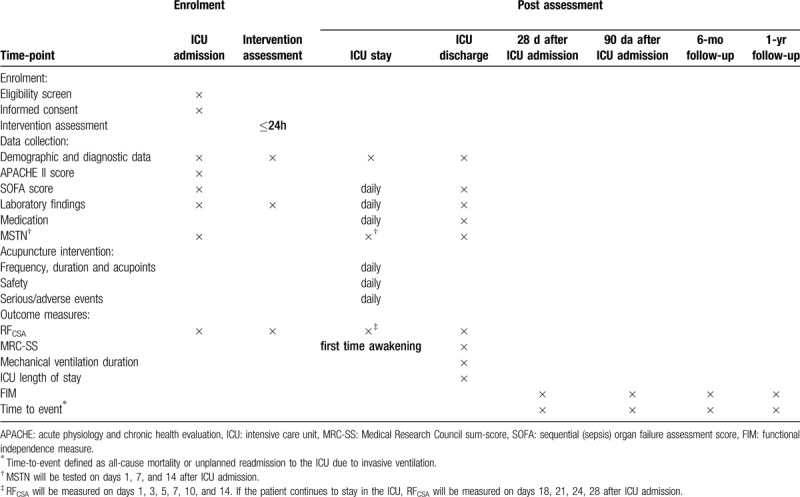
Participant timeline.

**Table 3:**
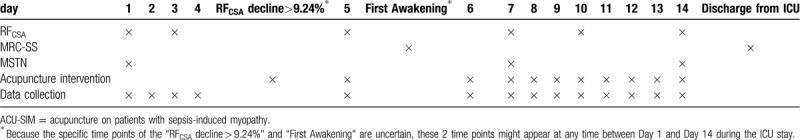
Bedside protocol for the ACU-SIM study.

#### 2.8.7 Patient follow-up

After the subject is discharged from the ICU, the researcher will not perform any form of intervention. According to the requirements of the observation indicators and their time points, follow-up data collection and filling will be performed 28 days, 90 days, 6 months and 1 year after ICU admission; patients will be followed until the follow-up time expires or the subject dies. The follow-up data collection includes the incidence of time-to-event, the FIM^TM^ scale and so on. Follow-up will be conducted by clinic follow-up and telephone follow-up. The investigator will notify the patient to return to the clinic for follow-up at a fixed time point. If it is inconvenient for the patient to go to the clinic, the follow-up will be conducted over the phone. If the patient refuses to proceed and asks to quit at any time during the follow-up, is the patient will be withdrawn from the study. If the patient cannot be contacted more than 3 times during the follow-up period, by any contact method (including telephone, mail, and various social software), is the patient will be considered lost to follow-up.

### 2.9 Recruitment

Ninety-eight subjects will be needed over the timeline of 2 years according to our sample size calculation. Within 24 hours of admission to the ICU, the patient will be screened against the inclusion criteria. The signing of an informed consent form for inclusion in the study will be performed once the patient or next-of-kin agrees to participate in the study. Data will be assessed only by the researchers, and all data will be kept confidential. Patient recruitment will begin in November 2019, and the expected completion date of the study is December 2022.

### 2.10 Statistical analysis

All available data will be analyzed descriptively. For continuous data, the normality test will be applied at the very beginning of the analysis. We will bewrite the results as the means, standard deviations, and 95% confidence intervals (CIs) for continuous data that conform to the normal distribution; medians, quartiles, and ranges for rank data and continuous data that do not fit the normal distribution; and percentages for discrete data.

The change in the RF_CSA_ from baseline will be analyzed by a generalized additive mixed model of repeated measures design. Analysis of the time-to-event outcome (at day 28, day 90, six months, and 1 year after the admission of ICU) will be conducted by applying Kaplan-Meier curves. Kaplan-Meier survival curves will be performed to describe the survival rate between the 2 groups, and the log-rank method will be used to compare the survival rates of the acupuncture group and control group. Multivariate Cox proportional hazard regression models will be used to analyze several constituents (such as acute physiology and chronic health evaluation-II, SOFA score, demographic and diagnostic data), adjust for covariates, and correct for confounding. Our team will use similar approach mentioned above to analyze secondary outcome measures when they can be expressed as time-to-event outcome dimensions. All regression analyses (generalized additive mixed models, log-rank analysis, multivariate Cox proportional hazard regression models) will include the same covariates as the primary analysis. If there are any measurable differences between the 2 study groups with regard to prognostic factors, a propensity score analysis will be applied to assess the sensitivity of the results to any treatment allocation biases. The propensity score will be set for the main outcome. After the 2 sets of data are analyzed according to the previous analysis, the patients will be matched with the propensity score, and then the main outcomes of the matched 2 groups will be compared again to see if there are any similarities or differences between the 2 groups before and after the match. If they are the same, the results are stable; however, a difference means that there are factors affecting the evaluation of the outcome before the match, and these factors will need to be explained.

### 2.11 Missing data

There are repeated measurement data (including RF_CSA_) in our research, and some data may be missing during the experiment. RF_CSA_ is 1 of the primary outcome indicators; if the baseline RF_CSA_ (the first measured value) is missing, no interpolation will be performed. In other situations, we will deploy a diagnostic analysis related to missing data. The ratio of missing data will be summed in each group and at each time point, and the threshold of this ratio is set to 5%. If the missing data is above the threshold, the Little test will be conducted. Otherwise, a specified primary and/or secondary outcome has less than 5% of missing data, a complete case analysis without interpolating the missing values. We will then analyze the complete cases without data imputation if the complete case value is implied to be a random missing by Little test. When Little's test betokens that the complete case data set is not a random missing, the threshold estimates and their 95% CIs will be proclaimed after implementing a worst- and best-case scenario imputation for the missing values. Multiple imputations (MI) will not be performed if the worst- and best-case analyses concede for the same conclusion. Oppositely, statistical software will create ten MI data sets beneath the hypothesis of data missing at random. After MI, our research team will pool the results of the trial of the intervention effect and 95% CI of the analyses of each data set.

### 2.12 Data monitoring

All data will be kept confidentially. Participants will be consigned with a code that will be adopted for all data management and analyses. The paper version of CRFs will be stored in a locked file cabinet. Coded electronic data will be stockpiled on a computer server at the First Affiliated Hospital of Guangzhou University of Chinese Medicine for the duration of the study. Entree to the computer records will be password-protected, and data will be accessible only to the investigation team. A data monitoring committee has been set up to conduct quality control assessments on the data. The completeness, consistency and plausibility of data will be supervised regularly and systematically and will be reported to the administration committee. The primary investigator (PI) will have access to the final deidentified data set. The reporting of adverse events, serious adverse events (SAEs) and suspected unexpected serious adverse reactions will appraise the safety of the study intervention. A descriptive analysis will be forwarded to the data monitoring committee regularly to assess the safety of the study intervention.

### 2.13 Adverse events

In this study, adverse events will be defined as follow:

(1) Fainting: Fainting means that the patient suddenly developed dizziness, palpitation, nausea, and even syncope during the acupuncture process; fainting is common in the acupuncture process.
(2) Broken needle: when the acupuncture needle breaks inside the patient.
(3) Hematoma: swelling and pain caused by a subcutaneous hemorrhage at the acupuncture site during or after the needle puncture.
(4) Bent needle: When the needle is inserted into the acupoint, the needle is bent in the body.
(5) Sticking of the needle: After the needle is inserted, when lifting and twisting the needle or when the needle is pulled out, the needle feels very heavy, tight, and even twitching; the needle is difficult to twist or remove, and the patient feels pain abnormal.
(6) Infection: infection at the acupuncture site after acupuncture.

If the above situations occur, they will be urgently handled by the acupuncturist and the ICU physician; if the researcher determines that the research plan does not needed to be adjusted, the research will continue. If the condition changes and the study treatment plan need to be stopped or changed, the subject will be withdrawn from the study and the events will be recorded in detail. All subsequent clinical treatments are based on the principle of saving the life of the subject and improving the prognosis of the disease. For all adverse events, the adverse event report form needs to be completed and the events need to be reported.

## 3 Discussion

SIM is a very serious complication that affects the recovery of septic patients.^[2]^ SIM progressively deteriorates in the early stage of the natural course of sepsis. Approximately 45% to 72% of sepsis patients in the ICU will have voluntary muscle atrophy,^[6,30,31]^ which could lead to a prolonged ICU LOS, unplanned reintubation and mechanical ventilation, unplanned readmission and increased mortality.^[7,8]^ Therefore, SIM has become an important risk factor for increasing mortality and disability of patients with sepsis.

The effects of sepsis on skeletal muscle are multifaceted. Studies have shown that active inflammatory reactions are an important cause of skeletal muscle dysfunction in sepsis.^[32,33]^ Its mechanism may be related to mitochondrial dysfunction caused by an inflammatory reaction.^[34,35]^ Meanwhile, the activity of the proteasome and lysosomal proteolytic system in sepsis patients is increased, leading to increased protein decomposition, which in turn affects skeletal muscle function and induces muscle weakness.^[36]^ MSTN is a new member of the transforming growth factor-β superfamily that has been discovered in recent years; MSTN mainly inhibits muscle growth and metabolism. High expression of MSTN can lead to muscle atrophy.^[11,37]^ In an animal experiment, acupuncture combined with low-frequency electrical stimulation downregulated the expression of MSTN and helped to alleviate muscle atrophy in mice.^[14]^ However, the in vivo expression level of MSTN in SIM patients and its relationship with SIM are not clear.

Previous studies have shown that muscle electrical stimulation therapy and physical therapy have some benefit on SIM.^[12,19,38]^ but the evidence to date is not solid.^[39,40]^ Acupuncture seems to be a promising approach to maintaining muscle mass in septic patients; nevertheless, clinical data specific to the efficacy of acupuncture on SIM are still lacking.

The research objectives of this prospective pCT are to provide preliminary data on the therapeutic, safety and long-term prognosis effects of acupuncture on SIM patients. We are looking forward to proposing a safe and effective method for the treatment of SIM.

## 4 Auditing

The study investigators will review the progress and data of the study each year. The Data Monitoring Committee will analyze and summarize the inclusion and withdrawal of research participants, data safety and other aspects.

## 5 Patient and public involvement

No patient or public involvement in the development of this study protocol.

## 6 Ethical approval and trial registration

The study will serve as a pilot trial of the upcoming real-world research project. This study has already been ratified by the Ethics Committee of the First Affiliated Hospital of Guangzhou University of Chinese Medicine (ZYYECK [2019] 130). In addition, this study has already been registered in the Chinese Clinical Trial Registry (Available at: http://www.chictr.org.cn/, No. ChiCTR-1900026308).

## 7 Consent or assent

Written informed consent will be obtained from each eligible patient or their next-of-kin at the very beginning of their first admission to the ICU and before any study procedure is performed, according to the Declaration of Helsinki.

This study will collect blood samples from patients for related tests. Residual blood samples will be kept in the Biomedical Research Library at Ling-Nan Medical Research Center.

## 8 Access to data

The final data set can only be viewed by the principal investigator. The acupuncturist, the outcome assessors, and other staff will only be able to view and record the data within their scope of work.

## 9 Confidentiality

This study will use CRFs to collect patient information. The completed CRF data will belong to the Department of Critical Care Medicine of the First Affiliated Hospital of Guangzhou University of Chinese Medicine. The data may not be provided to third parties under any circumstances without written permission.

## 10 Ancillary and posttrial care

If any adverse events occur in the later stage of the intervention, the clinician will take appropriate treatment measures according to the condition. The events and treatment will be recorded in detail.

## 11 Dissemination policy

The results of this study will be published to the public in the form of papers. The deidentified raw data can be obtained from the principal investigator within six months after the trial is completed.

## Acknowledgments

The authors would like to acknowledge and thank the staff of the acupuncture and intensive care departments at the First Affiliated Hospital of Guangzhou University of Chinese Medicine for their ongoing support of this project. And we also appreciated with Mr. Xu, Dang-Han's work in the design of the clinical study, made suggestions for the methodology and will perform the acupuncture.

## Author contributions

CWT is the primary investigator and responsible for the conception of the study design, planned the study, drafted the manuscript and checked the final draft of the manuscript. SC drafted and developed the manuscript and was involved in the design of this trial. ZYB, XDH, LDH and PZL will be responsible for conducting the study, data collection and evaluation. CJ made suggestions for the methodology and will make it possible to perform MRC-SS evaluation. XN helped in designing the clinical study and statistical analysis. LYY, HJ and TCZ contributed to developing the protocol, made suggestions for the methodology and checked the final draft of the manuscript. LXF, XNG, and XSX participated in the design, checked the final draft of the manuscript, and obtained funding for the study. LLM was responsible for the conception of the study design, developed the protocol, revised the manuscript, made suggestions for the methodology and checked the final draft of the manuscript. All authors went through and approved the final edition of the manuscript.
